# Quantifying the economic burden of unintended pregnancies due to drug–drug interactions with hormonal contraceptives from the United States payer perspective

**DOI:** 10.12688/gatesopenres.13430.1

**Published:** 2021-11-18

**Authors:** Meenakshi Srinivasan, Annesha White, Jason Lott, Todd Williamson, Sheldon X Kong, Leo Plouffe

**Affiliations:** 1 University of North Texas System College of Pharmacy, Fort Worth, TX, 76107, USA; 2 Bayer HealthCare Pharmaceuticals Inc, Whippany, NJ, 07981, USA

**Keywords:** drug-drug interactions, economic evaluation, hormonal contraceptives, unintended pregnancies, Markov model

## Abstract

**Background: **In the United States of America (USA), nearly 10 million women use oral contraceptives (OCs). Concomitant administration of certain medications can result in contraceptive failure, and consequently unintended pregnancies due to drug–drug interactions (DDIs). The objective of this analysis was to estimate the economic impact of unintended pregnancies due to DDIs among women of reproductive age using an OC alone or in combination with an enzyme inducer co-medication in the USA from a payer perspective.

**Methods: **A Markov model using a cohort of 1,000 reproductive-age women was developed to estimate costs due to contraceptive failure for OC alone *versus* OC with concomitant enzyme inducer drugs. All women were assumed to begin an initial state, continuing until experiencing an unintended pregnancy. Unintended pregnancies could result in birth, induced abortion, spontaneous abortion, or ectopic pregnancy. The cohort was analyzed over a time horizon of 1 year with a cycle length of 1 month. Estimates of costs and probabilities of unintended pregnancy outcomes were obtained from the literature. Probabilities from the Markov cohort trace was used to estimate number of pregnancy outcomes.

**Results:** On average, enzyme inducers resulted in 20 additional unintended pregnancies with additional unadjusted and adjusted costs median (range) of USD136,304 (USD57,436–USD320,093) and USD65,146 (USD28,491–USD162,635), respectively. The major component of the direct cost is attributed to the cost of births. Considering the full range of events, DDIs with enzyme inducers could result in 16–25 additional unintended pregnancies and total unadjusted and adjusted costs ranging between USD46,041 to USD399,121 and USD22,839 to USD202,788 respectively.

**Conclusion: **The direct costs associated with unintended pregnancies due to DDIs may be substantial and are potentially avoidable. Greater awareness of DDI risk with oral contraceptives among payers, physicians, pharmacists and patients may reduce unintended pregnancies in at-risk populations.

## Key points for policy makers

- Contraception failure due to drug-drug interactions (DDIs) with hormonal contraceptives may have a large public health implication due to the resulting unintended pregnancies. There is a lack of data regarding the clinical effect of DDIs affecting hormonal contraceptives which has been recognized by the FDA.
- Unintended pregnancies are a major economic burden to the US health care system. However, the economic impact of unintended pregnancies due to DDIs has not been estimated.
- We provide an estimate of the cost impact of unintended pregnancies due to DDIs using a Markov model.
- Greater awareness of the interacting potential among the numerous users of contraceptives and optimization of contraceptive prescribing policy by providers can lead to recognizing and consequently reducing this potentially avoidable economic burden for the payer.

## Introduction

Unintended pregnancies are a major economic burden, costing the United States (US) government USD21 billion in 2010 ^1^, and mainly result from contraceptive non-use or incorrect or inconsistent use of effective contraceptives ^2^. Several economic evaluations have established the cost-effectiveness of contraceptive use from a payer and societal perspective ^3– 5^. Long-acting reversible contraceptives (LARC), which include copper intrauterine device (IUD), levonorgestrel intrauterine system (IUS), and etonorgestrel implants have been found to be the most cost-effective options across various time-horizons and geographies ^3, 6– 16^. Although uptake of LARCs in the USA has been increasing, adoption has been low compared with oral contraceptives (OC), with 12% of reproductive age women using IUDs and 3% using implants compared to 25.3% using OCs in 2014 ^17^. With over 9.5 million women in the USA and 151 million women worldwide currently using OCs, factors leading to contraceptive failure and consequent unintended pregnancies among women constitute an important public health issue ^17– 19^. Non-adherence to OCs arising from forgetfulness, not filling a prescription, or experiencing side effects has been found to affect contraceptive efficacy ^20, 21^. The extent to which incorrect or inconsistent use of a contraceptive results in pregnancies, is quantified in a term known as the typical-use failure rate, while pregnancy rates during perfect use of a contraceptive are known as perfect-use failure rates, the former being a metric of contraceptive effectiveness in the real world, and the latter quantifying efficacy in a trial or controlled setting ^2^. Perfect-use or typical-use failure rates are quantitatively represented as the percentage of women experiencing an unintended pregnancy within the first year of use ^22^. Considering full adherence to OCs, another reason resulting in unintended pregnancies may be due to contraceptive failure from drug–drug interactions (DDIs).

Pharmacokinetic DDIs result with the co-administration of certain perpetrator drugs which may alter the systemic exposures of the OCs. Combined OCs consist of an estrogen component ( *e.g.* ethinyl estradiol) and a progestin component ( *e.g.* levonorgestrel, desogestrel, drospirenone) which are metabolized by cytochrome P450 enzymes in the liver to varying extents, depending upon their composition. Consequently, certain enzyme inducers may decrease the effectiveness of OCs and potentially result in unintended pregnancies, while certain enzyme inhibitors may increase plasma hormone concentrations, thereby increasing the incidence of adverse events such as thromboembolism ^23^. Drugs which interact with OCs and meant for long-term use include rifamycin antibiotics ^24^, antiepileptics ^25^, antiretrovirals ^26^ and psychotropic drugs ^27^, of which a few specific drugs have been classified by the CDC as Category 3, *i.e.*, the risks outweigh the advantages of using the method ^28^. Unintended pregnancies that occur as a consequence of DDIs are important, given that they occur in women who are otherwise compliant to their contraceptive method but who may be unaware of the impact of concomitant drugs on the effectiveness of their contraception.

Given the lack of data on the clinical effect of DDIs, the US Food and Drug Administration (FDA) uses class labelling for product labels of hormonal contraceptives based on information known about estrogens and progestins. To address the dearth of epidemiological data regarding the real-world impact of DDIs on unintended pregnancies a recent analysis of a commercial claims database by Sarayani *et al*. estimated the contraceptive failure rates among users of concomitant enzyme-inducer and enzyme-neutral antiepileptic drugs ^29^. The paucity of data regarding the clinical impact of DDIs on OCs was recognized by the FDA which resulted in a public meeting in 2015 which brought together regulators, clinicians and representatives from the industry and academia to seek solutions ^30^. The FDA recently released a guidance document for industry on clinical drug interaction studies for drugs under development having interacting potential with OCs and recommends alternative forms of contraception for women at risk of DDIs available at https://www.fda.gov/media/134581/download and https://www.fda.gov/media/110050/download. However, women may fail to receive sufficient counseling by their physician to make a switch to alternative contraception options, which puts them at risk of the consequences of DDIs such as unintended pregnancies or adverse events ^31, 32^. Currently, none of the economic evaluations address the economic impact of DDIs on hormonal contraceptives. The consequences of DDIs which may accrue substantial costs for the payer have not been addressed in economic evaluations. Therefore, the current study is a model-based analysis that aims to estimate the economic burden of unintended pregnancies due to DDIs with OCs in the USA from a payer perspective.

## Methods

### Overview

We developed a Markov model to estimate the effect of DDIs on OC effectiveness to estimate unintended pregnancies and total costs from a healthcare payer perspective. We compare three alternative strategies in the current model to characterize the excess costs attributable to DDI’s. Strategy 1 consists of OC use alone, therefore, the perfect-use failure rate for OCs is considered, which is 0.3% of women experiencing a pregnancy within the first year of use ( Table 1) ^2, 22^. For Strategies 2 and 3, which are OC failure in the presence of enzyme-inducer and enzyme-neutral drugs, respectively, we use real-world estimates from Sarayani *et al.* obtained from an analysis of a large commercial claims database ( Table 1) ^29^.

**Table 1.** Model cost and probability inputs.

Variable	Value	Reference
OC failure rates per 100 women-years		
Strategy 1: OC-alone	0.3	2, 22
Strategy 2: OC + enzyme-inducer drug (95% CI)	2.3 (1.9-2.8)	29
Strategy 3: OC + enzyme-neutral drug (95% CI)	1.6 (1.4-1.8)	29
**Probability estimates for unintended pregnancy outcomes**		
Birth	0.492	35– 39 ^†^
Induced abortion	0.350	
Spontaneous abortion	0.153	
Ectopic pregnancy	0.005	
**Cost of pregnancy outcomes (USD 2020) Median (Range)**		
Birth (unadjusted)	12,953 (5,270-28,664)	10, 13, 15, 40– 49 ^†^
Birth (adjusted)	5,497(2,290-12,453)	
Induced abortion	940 (601-4,233)	
Spontaneous abortion	1,121 (601-3,594)	
Ectopic pregnancy	6,174 (2,840-15,943)	

†Details regarding the derivation of these input parameters can be found in the supplementary materials OC: oral contraceptive

### Target population

The target population was a cohort of 1,000 “healthy” or “at risk”, sexually active, reproductive-aged US women aged 15–44 years using OCs. “Healthy” women were defined as those who take OCs alone (Strategy 1) without any interacting drugs and “at risk” women defined as those who have chronic co-morbid conditions requiring the use of interacting co-medications (Strategies 2 and 3, *e.g*., anti-epileptics, antiretrovirals, rifamycin antibiotics, *etc*.). All pregnancies occurring during the model time horizon were assumed to be unintended. As the intention of the current analysis was to estimate outcomes and cost of unintended pregnancies solely as a consequence of DDIs with OCs, contraceptive switching, discontinuation, or changes in pregnancy intentions were not modeled. We assume the same inherent average fertility in all women within the reproductive age group.

### Model design

**Figure 1.**
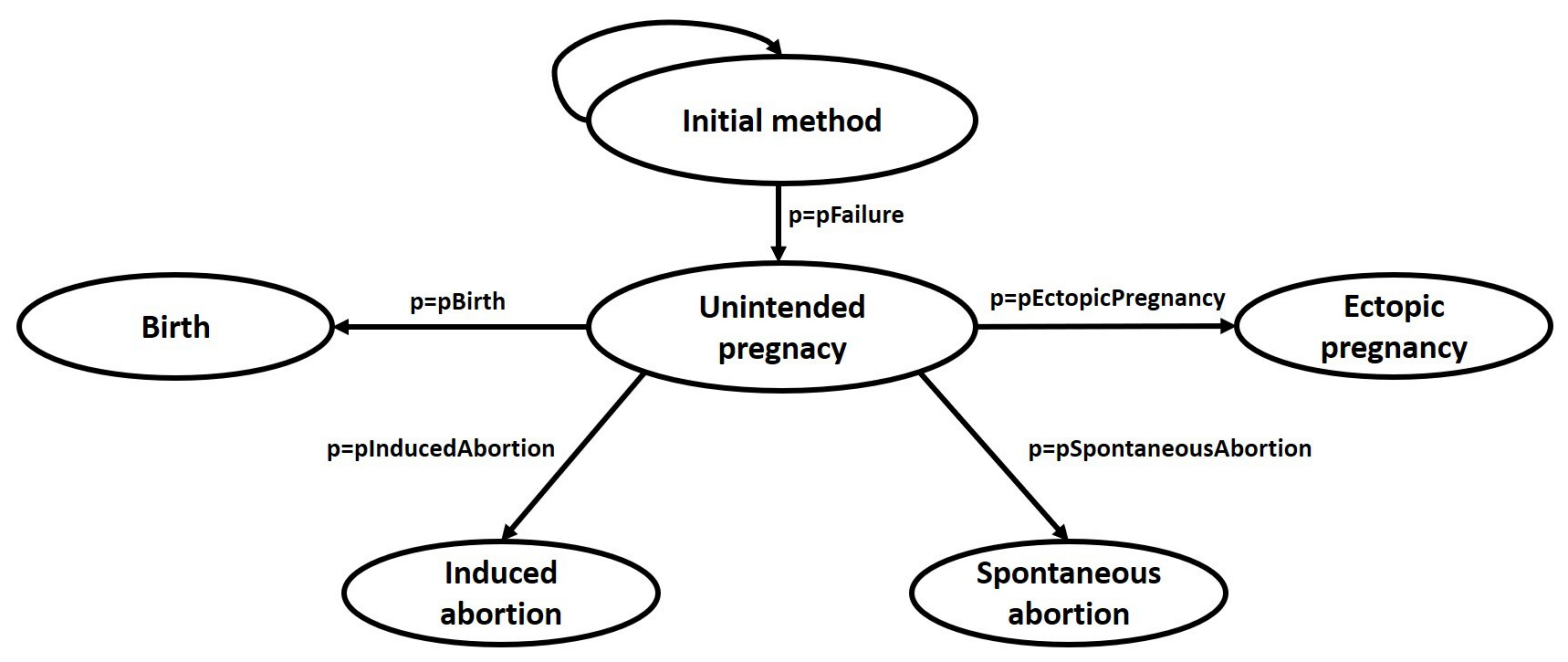
Model schematic. pFailure: transition probability of method failure; pBirth: transition probability of birth; pInducedAbortion: transition probability of induced abortion; pSpontaneousAbortion: transition probability of spontaneous abortion; pEctopicPregnancy: transition probability of ectopic pregnancy.

The model assumes a cycle length of one month and a time horizon of one year, since even when women are trying to conceive, the monthly fecundity rate is only 20% ^33^ (Age and Fertility. A Guide for Patients. American Society for Reproductive Medicine. 2012. https://www.reproductivefacts.org/globalassets/rf/news-and-publications/bookletsfact-sheets/english-fact-sheets-and-info-booklets/Age_and_Fertility.pdf) Our model comprises of five states. All women begin in the “initial method” (non-pregnant) state, consisting of either “OC alone” or “OC plus an enzyme inducer,” or “OC plus enzyme neutral drug” and continue in this state until experiencing contraceptive failure resulting in unintended pregnancy. Consequently, women may further transition to “birth”, “induced abortion”, “spontaneous abortion” or an “ectopic pregnancy”, which are assumed to be absorbing states. “Unintended pregnancy” by itself is not considered as a true state in the model and its outcomes have transition probabilities equal to the product of probability of OC failure and probability of unintended pregnancy resulting in the outcome ( *e.g*., probability of having a birth while on the pill is the product of pFailure and pBirth) ( Figure 1). After experiencing one of these terminal outcomes, a woman is assumed to discontinue the “initial method” and does not enter the model again.

### Transition probabilities

In order to delineate the effect of contraceptive failure due to DDI only, and not due to non-compliance of contraceptives, we assume 100% adherence to therapy and therefore consider perfect-use failure rates, *i.e*. Pearl index, defined as the number of failures per 100 woman–years of exposure for strategy 1 ^34^. The probability of OC failure during perfect use was obtained from Trussell *et al*. ^2, 22^. Yearly rates of method failure were converted to monthly estimates of probability using the following relationship; *p* = 1 – exp⁡[– *rt*] where *p* is the probability, *r* is the rate and *t* is the time period of interest ^50^. The probability estimates for unintended pregnancy outcomes are shown in Table 1. Number of pregnancies and pregnancy outcomes by age and proportion of births by intention status were obtained from the National Statistics reports ^35, 36^. The proportion of abortions by intention status were obtained from the literature ^37, 40, 41^. The age-wise distribution of ectopic pregnancies was obtained from Hoover *et al*. ^38^. Proportion of spontaneous abortions and ectopic pregnancies due to unintended pregnancies was assumed to be 45%, in accordance with the proportion of all pregnancies that were unintended ^39^. The derivation of probability inputs from these literature sources has been detailed in the Extended Data available at https://doi.org/10.5281/zenodo.5574442.

### Costs

We adopted the perspective of the healthcare payer, and therefore only direct medical costs of pregnancy outcomes were considered. Cost of the outcomes of unintended pregnancies included public and private payer perspectives ( Table 1) ^10, 13, 15, 40– 49^. All costs were adjusted to 2020 values to account for inflation using the medical component of the Consumer Price Index. The costs obtained from the various literature sources were summarized as median, minimum and maximum to reflect the central tendency, and range. Discounting was not conducted; however, the cost of live births was reduced to account for births that may have not been truly unwanted but simply mistimed. The fraction of unintended pregnancies that were mistimed ( *f*) were assumed to be 60% based on the age-wise distribution of unwanted and mistimed births ^36^, discount rate ( *r*) of 3% and the number of years the mistimed birth would have been delayed ( *d*) as 2 years ^51^, the adjusted cost is calculated as follows ^41^.

Costoflivebirth(adjusted)=Costoflivebirth(unadjusted)×(1−f(1+r)d)

The derivation of the cost inputs used in the model from the various literature sources has been detailed in the Extended Data available at https://doi.org/10.5281/zenodo.5574442.

### Scenario analysis

The uncertainty within the model was addressed through three scenario analyses to explore the robustness around the model assumptions:

- Scenario 1: The oral contraceptive failure rate was increased to consider the most recent estimate of typical-use OC failure rate of 7.2% ^52^. The failure rate for OC + enzyme inducer or OC + enzyme neutral drug, as obtained by Sarayani *et al*. ^29^ was consequently adjusted to account for the typical use failure rate as follows:

*Typical use failure rate for OC + enzyme inducer (enzyme-neutral) = Failure rate of OC + enzyme-inducer drug (enzyme–neutral drug) + (difference between typical and perfect use failure rate for OC alone)*

The impact of increasing the failure rate on unintended pregnancy outcomes and total adjusted cost of unintended pregnancies were quantified.

- Scenario 2: The abortion rate was increased to match the abortion rate in the US state with the highest percentage of unintended pregnancies ending in abortion, *i.e*., New York and decreased to match the abortion rate in the US state with the lowest percentage of unintended pregnancies ending in abortion, *i.e*., South Dakota ^53^. The impact of abortion rate on total number and adjusted costs of unintended births was quantified.
- Scenario 3: Pregnancy outcomes were adjusted to reflect age-specific patterns by adjusting the percentage of unintended pregnancies ending in abortion and birth while holding spontaneous abortion and ectopic pregnancy constant at base case levels. This was done for a fixed cohort size of 1000 and it was assumed that women are all from one age group at a time. The impact of age-specific pregnancy outcomes on total number and adjusted costs of unintended births was quantified.

### Analysis

The cumulative probability of the particular outcome at the end of the last cycle (cycle 12) in the Markov cohort trace was multiplied with the cohort size to obtain the expected number of events and the total costs for each outcome. Additional costs of the outcomes in strategy 2 ( *i.e*., “OC with concomitant enzyme-inducer use”) over the baseline ( *i.e*., strategy 1, “OC alone”) and additional costs of strategy 3 ( *i.e*., “OC with concomitant enzyme-neutral drug use”) over strategy 2 were then compared. The analysis was conducted on Microsoft Excel (RRID:SCR_016137; an open-access alternative is Google Sheets (RRID:SCR_017679)) and reproduced on R (v.3.6.3) (RRID:SCR_001905) based on a published coding framework ^54^ (Supplementary material available at https://doi.org/10.5281/zenodo.5574270).

### Model validation

Model validation was performed in alignment with current recommendations to ensure transparency and accuracy of the model applications ^55^. The model itself is being made available and is accessible to readers in the Extended Data. Face validity was performed by conducting an extensive literature search and curating an extensive database of relevant model input parameters and model structure information from all published economic evaluations conducted on hormonal contraceptives available in the public domain ^56^. Additionally, the sources of the model structure and input parameters were documented and reviewed by three independent health economic experts. Internal validity to ensure consistency and accuracy of model calculations were performed by double coding the model using spreadsheet software (Microsoft Excel) along with a script-based coding language (R v.3.6.3). In the absence of data regarding the outcomes of unintended pregnancy as a consequence of DDIs with OCs, external validation was limited; however, given the assumptions, the model outcomes were verified to ensure consistency with published contraception failure rates in each scenario. 

## Results

### Base-case analysis

The number and direct medical costs of UP accrued over a period of 1 year for a cohort of 1,000 women are shown in Table 2. On average, enzyme inducers resulted in 20 additional unintended pregnancies with additional unadjusted and adjusted costs of USD136,304 (USD57,436–USD320,093) and USD65,146 (USD28,491–USD162,635), respectively. The major component of the direct cost is attributed to the cost of births. Considering the full range of events, DDIs with enzyme inducers could result in 16 to 25 additional unintended pregnancies and total unadjusted and adjusted costs ranging between USD46,041 to USD399,121 and USD22,839 to USD202,788 respectively. Considering the real-world scenario, comparing concomitant use of OCs and enzyme-inducer drugs with OCs and enzyme neutral results in an average of 7 additional unintended pregnancies and additional unadjusted and adjusted costs ranging from USD14,309 to USD158,452 and USD7,098 to USD80,507 respectively.

**Table 2.** Number and cost of unintended pregnancies in various scenarios.

	Strategy 1	Strategy 2			Strategy 3		
Contraceptive failure rate per 100 women-years	0.3 (Estimate)	2.3 (Estimate)	1.9 (95% CI lower bound)	2.8 (95% CI upper bound)	1.6 (Estimate)	1.4 (95% CI lower bound)	1.8 (95% CI upper bound)
**Number of births (n)**	1	11	9	14	8	7	9
**Total cost of births (unadjusted) ** **(USD),** **median (range)**	19,090 (7,767-42,245)	144,903 (58,955-320,660)	119,942 (48,799-265,422)	175,966 (71,593 – 389,398)	101,155 (41,155-223,847)	88,599 (36,047-196,062)	113,686 (46,254 – 251,577)
**Total cost of births (adjusted)** ** (USD),** **median (range)**	8,293 (3,375 -18,353)	62,948 (25,618 – 139,310)	52,105 (21,205-115,312)	76,442 (31,109-169,173)	43,943 (17,883 – 97,250)	38,489 (15,664 – 85,179)	49,387 (20,099 – 109,297)
**Number of induced abortions (n)**	1	8	7	10	6	5	6
**Total cost of induced abortions ** **(USD),** **median (range)**	986 (630-4,438)	7,481 (4,783-33,687)	6,192 (3,959 – 27,884)	9,084 (5,808 – 40,908)	5,222 (3,339-23,516)	4,574 (2,924 – 20,597)	5,869 (3,752 – 26,429)
**Number of spontaneous** ** abortions (n)**	0	3	3	4	2	2	3
**Total cost of spontaneous** ** abortions (USD), median (range)**	514 (275-1,647)	3,900 (2,091 – 12,503)	3,228 (1,731-10,349)	4,736 (2,539 – 15,183)	2,722 (1,460-8,728)	2,384 (1,278-7,645)	3,060 (1,640 – 9,809)
**Number of ectopic pregnancies (n)**	0	0	0	0	0	0	0
**Total cost of ectopic pregnancies** ** (USD), median (range)**	92 (43-239)	702 (323 – 1,813)	581 (267-1,500)	852 (392-2201)	490 (225-1,265)	429 (197-1,108)	551 (253-1,422)
**Total number of unintended ** **pregnancies (n)**	3	23	19	28	16	14	18
**Total cost of unintended** ** pregnancies** **(unadjusted) (USD), median ** **(range)**	20,682 (8,715 – 48,569)	156,986 (66,151 – 368,662)	129,943 (54,756 – 305,155)	190,638 (80,332 – 447,690)	109,589 (46,179 – 257,357)	95,986 (40,447 – 225,412)	123,165 (51,900-289,238)
**Total cost of unintended ** **pregnancies** **(adjusted) (USD), median (range)**	9,885 (4,323-24,677)	75,031 (32,814 – 187,312)	62,106 (27,162-155,045)	91,115 (39,849-227,465)	52,378 (22,907 – 130,760)	45,876 (20,064 – 114,529)	58,866 (25,745 – 146,958)

Notes. Strategy 1 refers to perfect-use failure rate of oral contraceptive (OC) alone. Strategy 2 refers to OC failure rate with concomitant enzyme-inducer drugs and Strategy 3 refers to OC failure rate in the presence of concomitant enzyme neutral drugs. The first column of Strategy 2 and 3 represent the estimates considering the point estimate of the failure rate and the second and third column refer to the 95% CI lower bound and upper bound estimate, respectively. Pregnancy outcomes have been rounded to the nearest whole number; therefore, the sum of the pregnancy outcomes may not result in the total number of unintended pregnancies due to rounding. Additional costs of DDIs were calculated by subtracting estimates for Strategy 2 from Strategy 1.

### Scenario analysis

**Figure 2.**
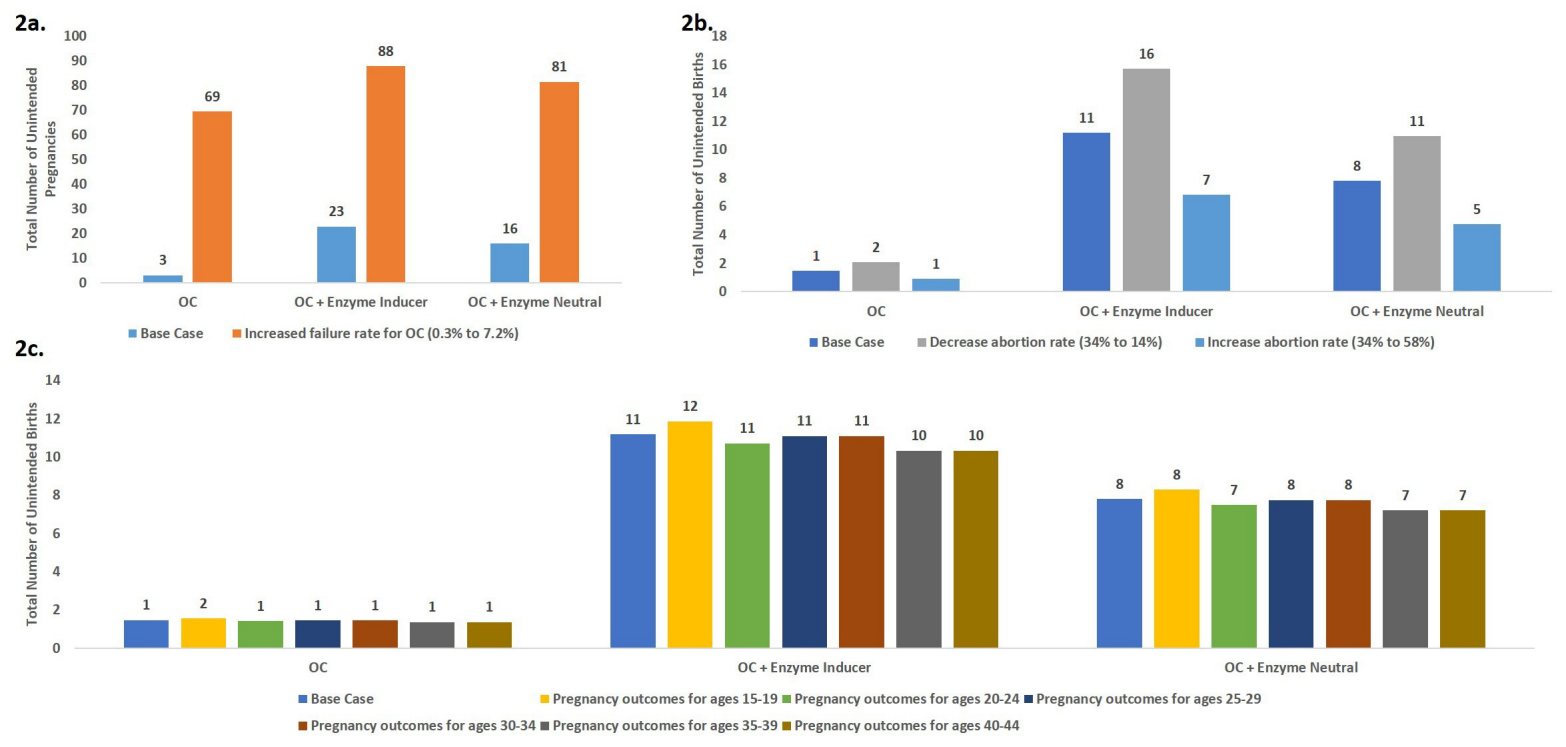
Scenario analysis: Comparison of key health outcomes for each scenario. Scenario 1. Increasing oral contraceptive failure rate; Scenario 2. Increased or Decreased Abortion Rate; Scenario 3. Age-specific patterns of pregnancy outcomes.

**Figure 3.**
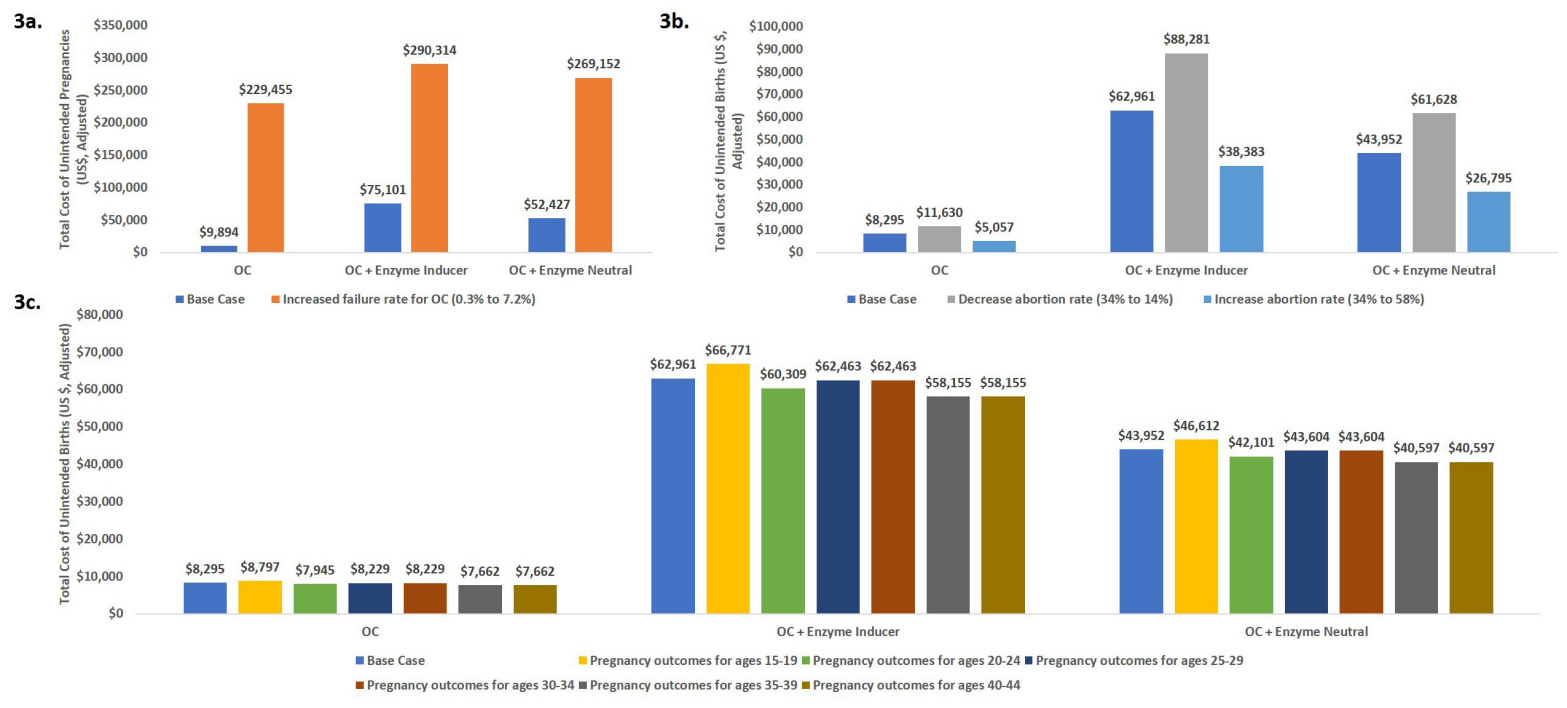
Scenario analysis: Comparison of key cost outcomes for each scenario. Scenario 1. Increasing oral contraceptive failure rate; Scenario 2. Increased or Decreased Abortion Rate; Scenario 3. Age-specific patterns of pregnancy outcomes.

Three scenario analyses were conducted to test the robustness of the model results and impact on pregnancy outcomes ( Figure 2) and cost ( Figure 3).

- Scenario 1: The model is highly sensitive to assumptions about the failure rates. Considering the typical-use failure rate resulted in 3.8-fold larger numbers of unintended pregnancies in the OC + enzyme-inducing drugs scenario as compared with the base-case scenario as shown in Figure 2a.
- Scenario 2: The model is sensitive to assumptions of abortion rates as shown in Figure 2b. The model predicted almost a 2-fold increase in number of unintended births when comparing the lowest and highest abortion rates.
- Scenario 3. The model was minimally sensitive to age-specific pregnancy outcomes as shown in Figure 2c.

## Discussion

The current analysis aimed to estimate the cost of UP attributed to DDIs with OCs for a cohort of 1,000 women considering various scenarios of contraceptive failure rates, namely perfect use with OC alone and in the presence of enzyme-inducer and enzyme-neutral drugs, estimates of which were obtained from a published real-world analysis. Given the large number of women who rely on OCs for their contraceptive needs in the USA and worldwide, even small incidences of these unintended pregnancies due to DDIs may result in substantial cost burden from a payer perspective. Therefore, interventions that seek to reduce care fragmentation and improve care coordination between primary care physicians and specialists will improve patient awareness and result in lower incidence of unintended pregnancy and adverse events because of DDIs. While clinical decision support systems embedded in electronic health record and pharmacy management software are designed to alert the physician or pharmacist of the potential of DDIs, these warnings are often overridden with rates as high as 90% due to “alert fatigue” ^57, 58^. In a recent US study of 255 pharmacies in the Chicago area, investigators found that about 52% of them dispensed medications without warnings about potential drug interactions, attributed to the emphasis on speed of filling prescriptions over quality among several retail pharmacies ^59^. Several approaches to reduce alert fatigue, such as classification of DDI’s based on priority are currently being evaluated to avert potential patient safety issues related to clinically significant drug interactions ^60^. Therefore, despite the presence of systems designed to identify and flag DDIs, several of these alerts can proceed unnoticed, potentially resulting in dangerous consequences to patients. In this context, we believe our study highlights the economic burden of one such important drug interaction and emphasizes on the need to provide additional safeguards in the drug prescribing and dispensing process. Dispensing OCs, whether from a retail pharmacy, online or through Planned Parenthood health centers should carefully consider the medication history, and offer sufficient counseling to women to ensure they are using contraception that is appropriate for their individual needs.

This is the first model-based analysis estimating the costs of DDIs with hormonal contraceptives. There have been only a few studies describing health services utilization costs associated with DDIs with antiretroviral therapy and opioids using real world data ^61– 66^. We considered a range of contraceptive failure rates, since the extent of enzyme induction and hence loss of efficacy of the OC can be dose dependent and drug specific, *e.g*., strong, moderate and weak CYP3A inducers decrease exposure of CYP3A substrates to different extents ^67^. We conducted the current analysis from a US payer perspective, the wide variation in the cost of the UP outcomes is reflective of costs accrued by public and private payers which were obtained from the published literature. The OC failure rates with enzyme-inducing and enzyme-neutral medications were calculated, assuming that drug exposure commenced at the prescription dispensing date and concluded on the last day of the pharmacy-entered dispensed days’ supply ^29^. However, this assumes women are fully adherent to their OC and likely underestimates failure rates for women who skip pills or discontinue use of their OC before the end of their pill supply. To account for this, we conducted a sensitivity analysis where we increased the OC-alone failure rate from the perfect (0.3%) to typical use (7.2%) failure rate and adjusted the concomitant enzyme-inducing and enzyme-neutral OC failure rates upward by a factor of 6.9% (7.2%–0.3%).

For each strategy within the total cohort, we assumed a constant failure rate which may not reflect real world experience due to heterogeneity in prescription medication and variation in DDI potential. The costs accrued due to adverse events with OCs which might be exacerbated by DDIs was not considered, as the extent to which adverse-event prevalence is altered in women taking interacting drugs is currently not well understood. Several costs were not considered in the current analysis, thus resulting in a likely underestimation of the true costs of DDIs. The direct medical costs of downstream events ( *e.g*., teratogenicity), events related to the reduced efficacy of the perpetrator drug, additional indirect costs due to work productivity losses, costs to social welfare programs and intangible costs were beyond the scope of the current analysis. The model also did not consider additional costs accrued due to OC discontinuation and switching. The impact of inter-individual variability on the clinical effect of DDIs by several pharmaceutical factors (dose, timing of drug administration, frequency, and duration of the specific interacting medication(s)) and non-pharmaceutical factors (timing of sexual intercourse with respect to menstrual cycle, use of back-up contraceptives) were not explored in the current analysis ( https://www.fda.gov/media/134581/download).

## Conclusions

The current study quantifies the consequences of a complex clinical pharmacology problem of DDIs with OCs which has large public health implications from an economic point-of-view. This question is currently being addressed by partners in academia and industry resulting in the integration of interdisciplinary fields of pharmacokinetic and pharmacodynamic modeling, pharmacoepidemiology and pharmacoeconomics ^30, 68, 69^. This cost represents a potentially avoidable and unrecognized economic burden to the payer, thus building a case for developing policy for population health decision making to optimize contraceptive prescribing and usage among women at risk of DDIs. Future iterations of this model can go beyond the current empiric estimation to incorporate variability in the clinical pharmacology in patients and consider additional costs and health states that may be expected due to DDIs.

## Data (and software) availability

### Underlying data

Zenodo: Underlying data for ‘Quantifying the economic burden of unintended pregnancies due to drug–drug interactions with hormonal contraceptives from the United States payer perspective’. https://doi.org/10.5281/zenodo.5574270

The project contains the following underlying data:

R codes and Microsoft Excel-based calculations related to manuscript: Quantifying the economic burden of unintended pregnancies due to drug-drug interactions with hormonal contraceptives from the United States payer perspective

This consists of R codes and Microsoft Excel based calculations of the pharmacoeconomic model described in the above referenced manuscript.

1. HCA DDI Markov model_Base Case.RMD: R codes for the calculation of the base case of the model

2. HCA DDI Markov model_SA1.RMD: R codes for the calculation of the Scenario Analysis 1 of the model

3. HCA DDI Markov model_SA2a.RMD: R codes for the calculation of the Scenario Analysis 2a of the model

4. HCA DDI Markov model_SA2b.RMD: R codes for the calculation of the Scenario Analysis 2b of the model

5. HCA DDI Markov model_SA3a: R codes for the calculation of the Scenario Analysis 3a of the model

6. HCA DDI Markov model_SA3b: R codes for the calculation of the Scenario Analysis 3b of the model

7. HCA DDI Markov model_SA3c3d: R codes for the calculation of the Scenario Analysis 3c and 3d of the model

8. HCA DDI Markov model_SA3e3f: R codes for the calculation of the Scenario Analysis 3e and 3f of the model

9. HCA DDI Markov model_Base Case.xlsx: Excel file for the calculation of the Base Case

10. HCA DDI Markov model_Scenario Analysis.xlsx: Excel file for the calculation of the Scenario Analyses

The derivation of the probability and cost input parameters have been described in the following file.

1. Derivation of probability and cost input parameters.docx available at https://doi.org/10.5281/zenodo.5574442

Data are available under the terms of the Creative Commons Zero "No rights reserved" data waiver (CC0 1.0 Public domain dedication).

### Reporting guidelines

Zenodo: CHEERS checklist for “Quantifying the economic burden of unintended pregnancies due to drug-drug interactions with hormonal contraceptives from the United States payer perspective”.

https://doi.org/10.5281/zenodo.5574430

Data are available under the terms of the Creative Commons Attribution 4.0 International (CC-BY 4.0).

## Consent

Not applicable. Study was based on aggregated data available from the literature.
