# Supplementary material for: Quantifying the economic burden of unintended pregnancies due to drug–drug interactions with hormonal contraceptives from the United States payer perspective
Source: Gates Open Res. 2021 Nov 18;5:171. [Version 1] doi: 10.12688/gatesopenres.13430.1 (PMC9722710; doi:10.12688/gatesopenres.13430.1)
Supplement: Peer review history

# Peer review: Reviewer response for version 1

**DOI:** 10.21956/gatesopenres.14685.r32744

**Published:** 2022-12-05

**Authors:** Aliza R. Karpes Matusevich

**Affiliations:** 1 School of Public Health, The University of Texas Health Science Center at Houston, Houston, TX, USA

The work is clearly and accurately presented but much of the supporting literature is not current. A quick PubMed check showed that this is likely due to lack of more recent studies on the topic. That said, it is possible that the rate of unintended pregnancies may be inflated as the source used examined a period in which rates of unintended pregnancies had increased in the United States – they have since decreased. Does your total of UP using OC failure rates correspond at all with the number of UP reported in Finer *et al.* (2016 ^1^)?

 In terms of the impact of DDIs on UP in the US – how many women of childbearing age, taking OC, are also taking enzyme inducers?

 It is a pity that authors could only rely on one study for OC failure rates due to DDI. I would have liked to have seen Sarayani’s conclusions summarized in the introduction.

 Is there any explanation for why users of enzyme-neutral drugs had more UP than women not taking medications?

 Could these results be inflated given that these women were likely not taking OCs perfectly? About 70% of women in Sarayani’s study had a bipolar disorder diagnosis and this could inflate rates of UP as rates of medication adherence for this group are only around 50% (Loots *et al.*, 2021 ^2^).

 Model:

- Is there justification for assuming the same fertility rate across the whole population?
- Was the mistimed rate also applied uniformly?
- Please provide more justification for the 60% discount in birth costs due to mistiming. It does not seem to correspond to the source literature.
- Scenario 3 seems to suggest that this would not have a large impact on results, but I would like these parameters to be addressed explicitly.
- Was this a cohort or a probabilistic model – based on results, I would presume cohort?
- A larger cohort is recommended.
- Was the cohort stratified by age or marital status?
- What was distribution of age among the population – same as whole population or same as population on OC?
- Overall, I would recommend reporting using CHEERS or other standard for reporting on CEA
- Interesting discussion on why physicians might not be informing women on the possibility of DDIs – room for intervention in this area.
- Excellent discussion of limitations.

Is the work clearly and accurately presented and does it cite the current literature?

Partly

If applicable, is the statistical analysis and its interpretation appropriate?

Yes

Are all the source data underlying the results available to ensure full reproducibility?

Yes

Is the study design appropriate and is the work technically sound?

Yes

Are the conclusions drawn adequately supported by the results?

Yes

Are sufficient details of methods and analysis provided to allow replication by others?

Yes

Reviewer Expertise:

Health Economics and Outcomes Research

I confirm that I have read this submission and believe that I have an appropriate level of expertise to confirm that it is of an acceptable scientific standard, however I have significant reservations, as outlined above.

## References

[1] : Declines in Unintended Pregnancy in the United States, 2008-2011. *N Engl J Med* .2016;374(9) : 10.1056/NEJMsa1506575 843-52 10.1056/NEJMsa1506575 26962904PMC4861155

[2] : Interventions to Improve Medication Adherence in Patients with Schizophrenia or Bipolar Disorders: A Systematic Review and Meta-Analysis. *Int J Environ Res Public Health* .2021;18(19) : 10.3390/ijerph181910213 10.3390/ijerph181910213 34639510PMC8508496

# Peer review: Reviewer response for version 1

**DOI:** 10.21956/gatesopenres.14685.r32756

**Published:** 2022-11-14

**Authors:** Luis Bahamondes

**Affiliations:** 1 Department of Obstetrics and Gynecology, State University of Campinas, Campinas, Brazil

Meenakshi Srinivasan and co-workers submitted a manuscript entitled: “Quantifying the economic burden of unintended pregnancies due to drug–drug interactions with hormonal contraceptives from the United States payer perspective”.

 This is a well conducted study and well-written manuscript which explores an interesting and neglected issue.

 OC is the most prevalent contraceptive method worldwide with few exceptions and consequently the assessment of economic burden of unintended pregnancies due to drug-drug interaction during OC intake is extremely important, not only for healthcare providers but also for users and potential users. The analysis of 1-year horizon is appropriate. The authors found that the use of enzyme inducers resulted in 16-25 additional unintended pregnancies. These findings reveal a high cost for the health system including the cost of antenatal care, delivery, postnatal care, and childcare. It is important and provides clear guidance to providers and policy makers.

Is the work clearly and accurately presented and does it cite the current literature?

Yes

If applicable, is the statistical analysis and its interpretation appropriate?

Yes

Are all the source data underlying the results available to ensure full reproducibility?

Yes

Is the study design appropriate and is the work technically sound?

Yes

Are the conclusions drawn adequately supported by the results?

Yes

Are sufficient details of methods and analysis provided to allow replication by others?

Yes

Reviewer Expertise:

NA

I confirm that I have read this submission and believe that I have an appropriate level of expertise to confirm that it is of an acceptable scientific standard.

# Peer review: Reviewer response for version 1

**DOI:** 10.21956/gatesopenres.14685.r32143

**Published:** 2022-06-22

**Authors:** Evan Myers

**Affiliations:** 1 Department of Obstetrics and Gynecology, Duke University School of Medicine, Durham, NC, USA

Overall, a potentially useful analysis to help guide policy makers and researchers about the potential economic impact of drug-drug interactions (DDIs) on contraceptive effectiveness. Although the presentation is clear and the results broadly intuitive, there are a number of issues which should be clarified/addressed.

**Introduction:**

 The context of the research is in general clearly described, but there is one key item that is missing: although no one would disagree that "unintended pregnancies that occur as a consequence of DDIs are important", but what proportion of unintended pregnancies are attributable to DDI vs perfect use vs imperfect use?  Reference 29 provides some data on the relative risk for one class of drug. How many women of reproductive age are prescribed drugs with potential DDIs? If one assumes that the age-specific distribution of contraceptive methods is similar compared to women not using these drugs (a generous assumption), what is a plausible range for the number of women at risk? Given relative risk estimates in reference 29, the number of unintended pregnancies? How does this compare to unintended pregnancies from other causes of OC failure?  

**Methods:**

1. Target population: 
  a) Why a cohort of only 1000? From a modeling perspective, this potentially limits the ability to incorporate probabilistic approaches (which would be appropriate here). If the rationale is that a typical payer would only have 1000 reproductive aged women using OCs and enzyme inducers, this needs to be explicitly stated. The estimation of potential number of women to whom the analysis would apply discussed above should inform this decision. 
  b) I'm not sure using "healthy" women as the comparator is appropriate, certainly not with the same size "cohort". If the purpose is to model relative economic burden, then "Strategy 1" is really a comparative population group, and the size of the cohort (and age distribution with all that implies for fecundity and pregnancy outcomes) should be substantially larger. The assumption of the "same inherent average fertility in all women within the reproductive age group" isn't really justifiable here, since both age distribution and the effects of the co-morbid conditions may affect both fecundity and the likelihood of outcomes (including both induced and spontaneous abortion, as well as the cost of livebirth given that most of these would be, by definition, high-risk pregnancies. Since reference 29 provides an estimate of the impact of DDIs compared to non-inducing drugs on unintended pregnancies, why not just model that?  
2. Model:
  a) A monthly cycle length is reasonable, but I would think the time horizon should be at least two years since many of the births wouldn't occur until the second year.  
  b) Cycle fecundity changes substantially after age 35; the age distribution of women using drugs with potential DDIs is important (another reason why the use of "healthy" women aged 15-44 is not an appropriate comparator without specific justification). If accounting for age distribution is not included, the authors need to provide a justification and a discussion of the implications.  
3. Transition probabilities:
  a) Again, spontaneous abortion rates increase dramatically with age, and the underlying population distribution for women with the comorbid conditions of interest should be used.  
  b) Similarly, depending on the underlying condition, induced abortion rates may be substantially higher because of the potential risks of the underlying condition on maternal morbidity/mortality, and/or associations between drugs taken for the condition and adverse embryonic/fetal/neonatal outcomes.
4. Costs:
  Because, by definition, all of these pregnancies will be high-risk, the costs of live births is underestimated (and, since risk of preterm birth is increased, lifetime costs for infants will be underestimated as well). This should be acknowledged and discussed.
5. Scenario Analysis:
  a) Scenario 1 (typical use) is much more appropriate than the current base case. The paper would be much stronger if typical use was the base case and "perfect use" an alternative scenario.
  b) Scenario 3 is not valid without changing age-specific spontaneous loss for age (Magnus *et al.*, 2019) ^1^ or age-specific fecundity. The results shown in Figures 2c and 3c do not meet basic face validity--there is no way that the expected number of pregnancies over one year in 1000 women aged 40-44 is equivalent to the number in women 20-24.   
6. Additional:
  Why not do a probabilistic sensitivity analysis as well, given that appropriate distributions for many of these parameters are available?  

**Results:**

1. Again, I fail to see any value to the comparison to "healthy" women.  
2. Given that, why are failure rates for women using enzyme neutral drugs so much higher than for "healthy" women? I suspect it is because the assumption of perfect use for Strategy 1 and that the data for Strategy 3 come from real-world data, but if that's the case, then Scenario 1 should be the base case comparator. Even in that scenario, higher failure rates in women with a likely older age distribution and chronic conditions that may affect fecundity (or at least coital frequency) has face validity issues.

**Discussion:**

 The points raised about limitations are all relevant and are reasonable (particularly the potential sources of underestimation of costs), but I believe the issues raised above are much more limiting and need to be addressed.

Is the work clearly and accurately presented and does it cite the current literature?

Yes

If applicable, is the statistical analysis and its interpretation appropriate?

Partly

Are all the source data underlying the results available to ensure full reproducibility?

Yes

Is the study design appropriate and is the work technically sound?

Partly

Are the conclusions drawn adequately supported by the results?

Partly

Are sufficient details of methods and analysis provided to allow replication by others?

Yes

Reviewer Expertise:

I am trained as a general obstetrician/gynecologist and epidemiologist, with extensive experience in modeling and economic analysis, including modeling of fertility and pregnancy outcomes.

I confirm that I have read this submission and believe that I have an appropriate level of expertise to confirm that it is of an acceptable scientific standard, however I have significant reservations, as outlined above.

## References

[1] : Role of maternal age and pregnancy history in risk of miscarriage: prospective register based study. *BMJ* .2019; 10.1136/bmj.l869 10.1136/bmj.l869 PMC642545530894356
